# C-Terminal Mutants of Apolipoprotein L-I Efficiently Kill Both *Trypanosoma brucei brucei* and *Trypanosoma brucei rhodesiense*

**DOI:** 10.1371/journal.ppat.1000685

**Published:** 2009-12-04

**Authors:** Laurence Lecordier, Benoit Vanhollebeke, Philippe Poelvoorde, Patricia Tebabi, Françoise Paturiaux-Hanocq, Fabienne Andris, Laurence Lins, Etienne Pays

**Affiliations:** 1 Laboratory of Molecular Parasitology, IBMM, Université Libre de Bruxelles, Gosselies, Belgium; 2 Laboratory of Animal Physiology, IBMM, Université Libre de Bruxelles, Gosselies, Belgium; 3 Centre de Biophysique Moléculaire Numérique, Université de Gembloux, Gembloux, Belgium; University of Wisconsin-Madison, United States of America

## Abstract

Apolipoprotein L-I (apoL1) is a human-specific serum protein that kills *Trypanosoma brucei* through ionic pore formation in endosomal membranes of the parasite. The *T. brucei* subspecies *rhodesiense* and *gambiense* resist this lytic activity and can infect humans, causing sleeping sickness. In the case of *T. b. rhodesiense*, resistance to lysis involves interaction of the Serum Resistance-Associated (SRA) protein with the C-terminal helix of apoL1. We undertook a mutational and deletional analysis of the C-terminal helix of apoL1 to investigate the linkage between interaction with SRA and lytic potential for different *T. brucei* subspecies. We confirm that the C-terminal helix is the SRA-interacting domain. Although in *E. coli* this domain was dispensable for ionic pore-forming activity, its interaction with SRA resulted in inhibition of this activity. Different mutations affecting the C-terminal helix reduced the interaction of apoL1 with SRA. However, mutants in the L370-L392 leucine zipper also lost *in vitro* trypanolytic activity. Truncating and/or mutating the C-terminal sequence of human apoL1 like that of apoL1-like sequences of *Papio anubis* resulted in both loss of interaction with SRA and acquired ability to efficiently kill human serum-resistant *T. b. rhodesiense* parasites, *in vitro* as well as in transgenic mice. These findings demonstrate that SRA interaction with the C-terminal helix of apoL1 inhibits its pore-forming activity and determines resistance of *T. b. rhodesiense* to human serum. In addition, they provide a possible explanation for the ability of *Papio* serum to kill *T. b. rhodesiense*, and offer a perspective to generate transgenic cattle resistant to both *T. b. brucei* and *T. b. rhodesiense*.

## Author Summary

The serum protein apolipoprotein L-I (apoL1) is responsible for human innate immunity against *Trypanosoma brucei brucei*, because this protein kills the parasite by generating ionic pores in the lysosomal membrane. Two *T. brucei* subspecies (*T. b. rhodesiense* and *T. b. gambiense*) can resist apoL1 and therefore, infect humans and cause sleeping sickness. In *T. b. rhodesiense*, resistance to human serum is linked to interaction of the Serum Resistance-Associated (SRA) protein with the C-terminal region of apoL1. We show that mutations targeted to this region reduced its interaction with SRA while preserving the activity of the ionic pore-forming domain. While some mutants also lost their trypanolytic potential, C-terminal mutants inspired by apoL1-like sequences of *Papio anubis* conserved this activity, but also acquired the ability to efficiently kill *T. b. rhodesiense*, both *in vitro* and in mice. These findings demonstrate that interaction of SRA with the C-terminus of apoL1 inactivate this protein and is responsible for the resistance of *T. b. rhodesiense* to human serum. Moreover, they suggest that apoL1-like proteins could be responsible for the trypanolytic potential of *Papio* species. Finally, *Papio*-like human apoL1 mutants could be used to generate transgenic cattle that would resist both *T. b. brucei* and *T. b. rhodesiense*.

## Introduction

Normal human serum (NHS) is able to kill *T. b. brucei*, but not *T. b. rhodesiense* and *T. b. gambiense*. The lytic factor was identified as being apoL1 [1],[2]. This protein is associated with HDL particles that are efficiently taken up by the parasite through specific binding to a haptoglobin-hemoglobin surface receptor, due to the simultaneous presence of haptoglobin-related protein (Hpr) acting as a ligand in these particles [3]. Trypanosome lysis results from anionic pore formation by apoL1 in the lysosomal membrane of the parasite [4]. Resistance to lysis has only been studied in case of *T. b. rhodesiense*, where it was shown to depend on a parasite protein termed SRA [5]. As synthesis of SRA only occurs after transcriptional activation of a given Variant Specific Glycoprotein (VSG) gene expression site from a repertoire of 10–20 candidates, *T. b. rhodesiense* clones can be either sensitive or resistant to NHS depending on which expression site is active [2],[5]. The mechanism by which SRA inhibits the activity of apoL1 is unclear. Direct coil-coiling interaction between the C-terminal α-helix of apoL1 and the N-terminal α-helix of SRA was demonstrated *in vitro*, but *in vivo* only evidence for tight co-localization between the two proteins was obtained [1]. Total deletion of the C-terminal helix appeared to confer toxic activity to recombinant apoL1 on *T. b. rhodesiense*, suggesting that *in vivo* SRA neutralizes apoL1 through interaction with its C-terminal domain [1]. However, the trypanolytic effect of the deleted apoL1 was weak and incomplete [1]. Moreover, data obtained following transgenic expression of a similarly truncated apoL1 in mice suggested that its trypanolytic potential was lost *in vivo* [8]. Therefore, additional work was required to evaluate if the interaction observed *in vitro* was relevant for the *in vivo* mechanism of *T. b. rhodesiense* resistance to human serum.

While human serum is unable to kill *T. b. rhodesiense*, the serum of some African primates like *Papio sp*. was equally active on both *T. b. rhodesiense* and *T. b. brucei* [6],[7]. The phenotype of trypanolysis by *Papio* serum strikingly resembled that induced by human serum, as it was also dependent on HDL particles and was similarly inhibited by competing amounts of haptoglobin [7]. Therefore, it could be envisaged that in *Papio*, an apoL1-like protein unable to interact with SRA would be responsible for the trypanolytic activity of these primates. We used this working hypothesis as a base to construct human apoL1 mutants.

We analyzed the effects of various deletions and mutations in the C-terminal domain of apoL1 on the trypanolytic potential of this protein against *T. b. brucei* and *T. b. rhodesiense*. The results confirmed the interaction model presented in [1], as well as the role of this interaction in resistance to apoL1 in *T. b. rhodesiense*. In accordance with these findings, *Papio*-like apoL1 mutants able to efficiently kill both *T. b. brucei* and *T. b. rhodesiense* were identified.

## Results

### Direct interaction of SRA with apoL1 inactivates the pore-forming activity of this protein

**Figure 1:**
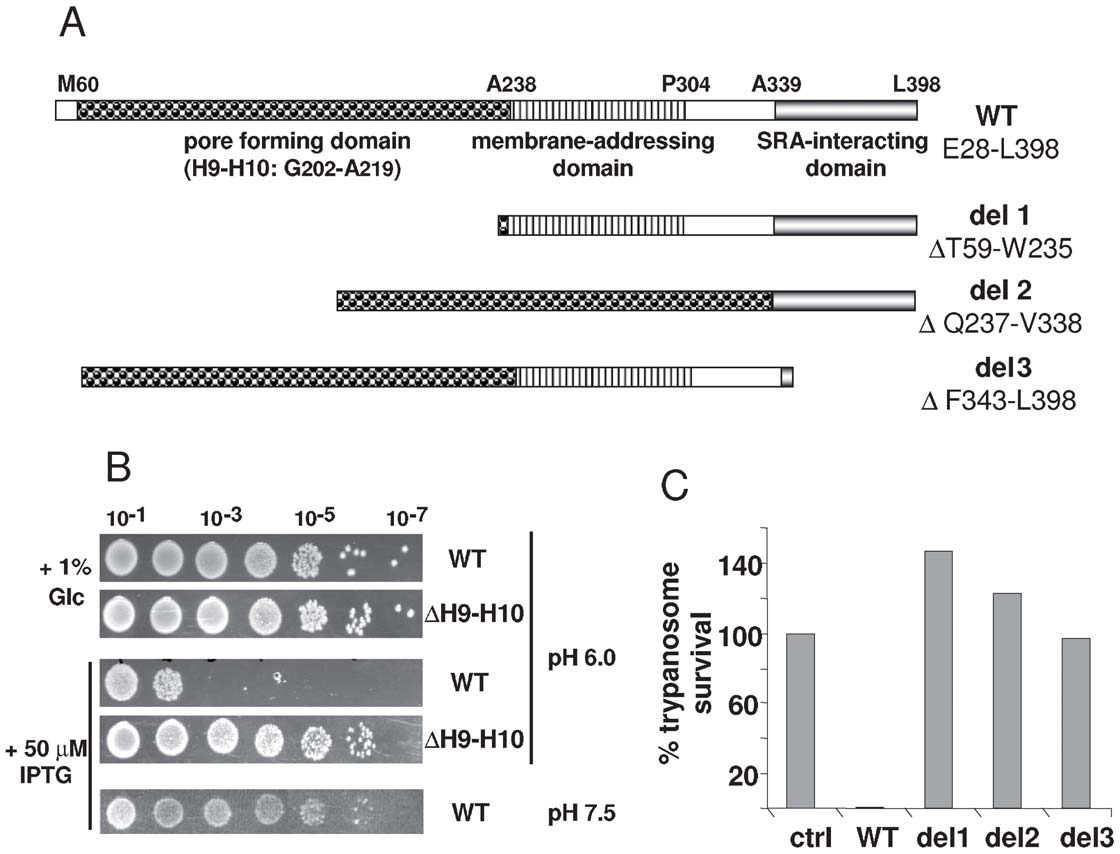
Lytic activities of apoL1. **A.** Schematic representation of apoL1 and apoL1 variants analyzed in this work, with indication of the amino acid residues delineating the three protein domains (see ref. 4 for details). **B.** Colicin-like activity of WT and mutant apoL1. pCDF-DUET plasmids encoding apoL1 variants provided with a C-terminal V5-His6 tag were transfected into *E. coli* BL21(DE3). In the case of the deletion (del) variants, an N-terminal bacterial signal peptide (pelB) was added. After overnight incubation at 37°C the bacterial plating efficiency was scored comparing expression induction by IPTG addition or non-induction by glucose (Glc) addition, or comparing the effect of pH. The ΔH9–H10 apoL1 mutant lacks the region encoding helices 9 and 10 of the pore-forming domain. **C.** Trypanolytic potential of various apoL1 variants, as determined on *T. b. brucei* after 24 h–incubation *in vitro* (ctrl = control without apoL1).

As illustrated in Fig. 1A, apoL1 contains three functional domains responsible for its ionic pore-forming capacity, addressing to biological membranes and interaction with SRA, from N- to C-terminus respectively [4].

In *E. coli*, the pore-forming activity of apoL1 can be measured by the reduction of cell viability following IPTG-induced expression of the apoL1 gene from plasmid constructs [4]. In this system apoL1 expression closely mimicked the toxicity of the isolated pore-forming domain of bacterial colicin A fused to the signal peptide pelB, including depolarization of the plasma membrane [4]. As a control, deletion of the essential helix 9 of the pore-forming domain completely prevented this activity (Fig. 1B). Although the pore-forming domain of apoL1 appeared to have access to the inner membrane of *E. coli*, it did not appear to be secreted as neither apoL1-expressing cells nor culture medium from these cells killed wild type *E. coli* when mixed with these cells (data not shown). The toxicity exhibited by the apoL1 pore-forming domain was clearly dependent on acidic pH, since it occurred when *E. coli* was grown in LB medium at pH 6, but not at pH 7.5 (Fig. 1B).

**Figure 2:**
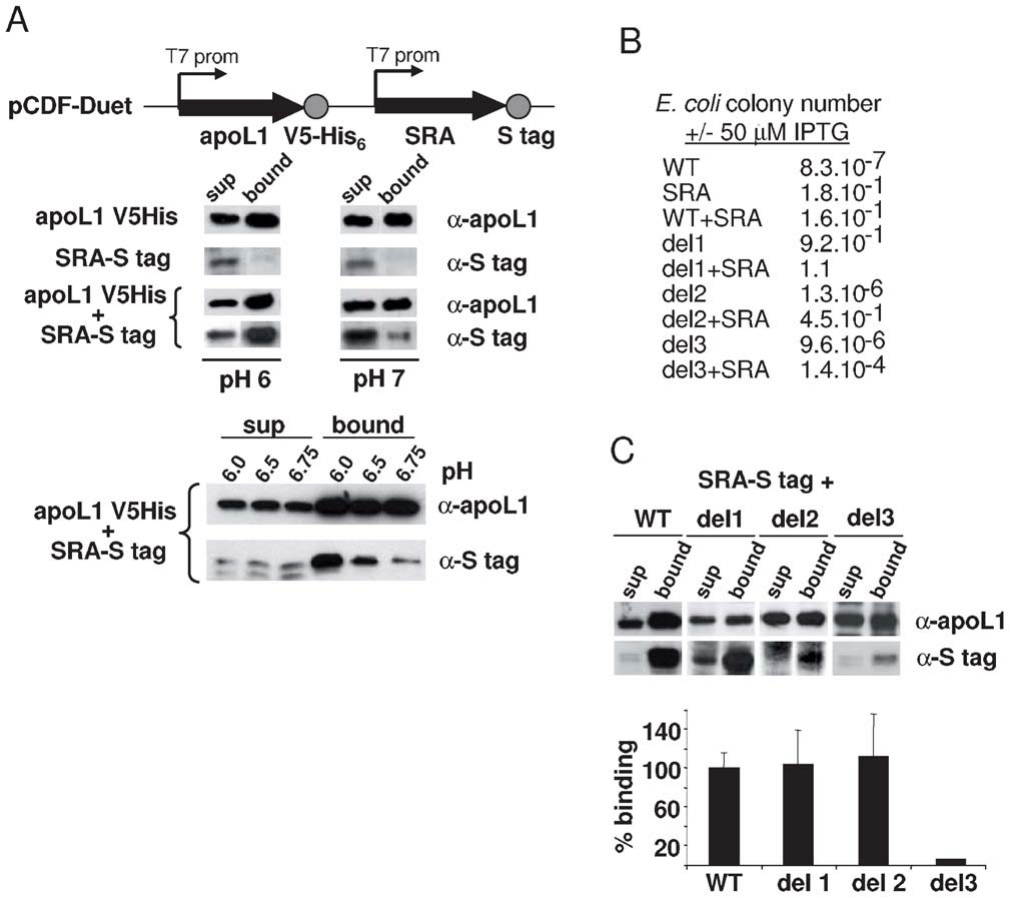
SRA interaction with apoL1. **A.** Double expression of apoL1 and SRA in *E. coli*. The scheme of the plasmid construct is shown above Western blot data illustrating the detection of the two tagged proteins from the lysis supernatant (sup = supernatant) and their recovery in the fractions bound to nickel beads. ApoL1 and SRA were revealed by anti-apoL1 and anti-S tag antibodies, respectively. The pH of the extraction buffer was between 6 and 7, as indicated. **B.** Influence of apoL1 and/or SRA expression on bacterial plating efficiency determined by the ratio of colonies counted following induction *versus* non-induced controls, as illustrated in Fig. 1B. **C.** Evaluation of the level of protein association with the nickel beads. SRA binding to apoL1 was expressed as SRA binding percentage divided by the percentage of apoL1 binding to the nickel beads, which varied depending on the type of mutation/deletion performed on the protein. This ratio was considered as 100% for SRA binding to WT apoL1. In *E. coli* co-expressing WT apoL1 and SRA, the typical yield of each protein was respectively 3 µg and 100 ng/10^10^ cells. In this and the following figures, the values resulted from three independent experiments each performed in triplicate.

In addition to its effect on *E. coli*, the pore-forming activity of apoL1 is also responsible for the ability of this protein to kill trypanosomes, although the trafficking and intracellular targeting of the toxin are obviously very different between the two systems [4]. Addition of recombinant apoL1 to *T. b. brucei* resulted in efficient killing of the parasite, as measured after overnight incubation (Fig. 1C). The trypanolytic activity of apoL1 is known to be inhibited by the *T. b. rhodesiense* protein SRA [1],[5]. In order to analyze the mechanism by which SRA neutralizes apoL1 pore-forming activity, we constructed a bicistronic plasmid co-expressing the two proteins in *E. coli*, under the dependence of two inducible T7 transcription promoters (pCDF-DUET; Fig. 2A). In this system apoL1 was tagged at the C-terminus with V5 and 6-His peptides, whereas SRA was provided with a C-term S tag. Following induction of expression, apoL1 was purified by binding to nickel beads, and after elution it was revealed using anti-apoL1 antibodies. As shown in Fig. 2A, apoL1 was clearly detected in the nickel-bound fraction. When co-expressed with apoL1, SRA was also present in this fraction as revealed by anti-S tag antibodies. However, in the absence of apoL1, SRA was no longer found in the bound fraction (Fig. 2A). These results indicated that in *E. coli*, co-expression of apoL1 and SRA results in the formation of a complex associating these two proteins. Consistent with the lysosomal localization of this complex in *T. brucei* [1], the formation of the apoL1-SRA complex appeared to be favoured at acidic pH as its amount was strongly reduced upon *E. coli* lysis at different pH values between pH 6 and pH 7 (Fig. 2A).

Significantly, the pore-forming activity of apoL1 was strongly inhibited upon co-expression of SRA (Fig. 2B). Therefore, in the *E. coli* expression system apoL1 appeared to be inactivated following direct interaction with SRA.

### The C-terminal domain of apoL1 is entirely responsible for the interaction of this protein with SRA

We evaluated the level of interaction of SRA with different mutants of apoL1 by measuring the relative amounts of either protein bound to nickel beads. More precise measurement of this interaction using plasmon resonance was impossible, due to the propensity of both proteins to stick to various matrices (data not shown). We generated apoL1 variants deleted of either one of the three functional domains (del 1, del 2 and del 3 from N- to C-term, see Fig. 1A). The presence in each case of an N-terminal bacterial signal peptide (pelB) allowed the determination of the pore-forming potential of the variants in *E. coli* irrespective of the deletion of the membrane-addressing domain [4]. As expected [4], only deletion of the N-terminal domain (del 1) resulted in the loss of the pore-forming activity (Fig. 2B). Co-expression of SRA with del 2 resulted in strong inhibition of the killing activity like it was observed with wild-type apoL1, whereas SRA only mildly affected the activity of del 3 (Fig. 2B). In accordance with these data, SRA was found to bind to del 1 and del 2, but not to del 3 (Fig. 2C). Altogether these data confirmed that SRA interacts with the apoL1 C-terminal domain, and revealed that this interaction inhibits apoL1 activity independently of the presence of the membrane-addressing domain.

### Deletion or mutations of the C-terminal domain do not affect ionic pore-formation, but impede trypanolytic activity

Recombinant forms of the three apoL1 variants del 1, del 2 and del 3 were produced in *E. coli* and tested for their trypanolytic potential. As expected, del 1 and del 2 were inactive (Fig. 1C). Surprisingly, despite the full conservation of its intrinsic pore-forming activity (Fig. 2B), del 3 was also inactive (Fig. 1C). These findings confirmed a recent report [8], but contrasted with our previous work where recombinant del 3 expressed in Chinese hamster ovary cells was found to kill NHS-sensitive trypanosomes [1].

**Figure 3:**
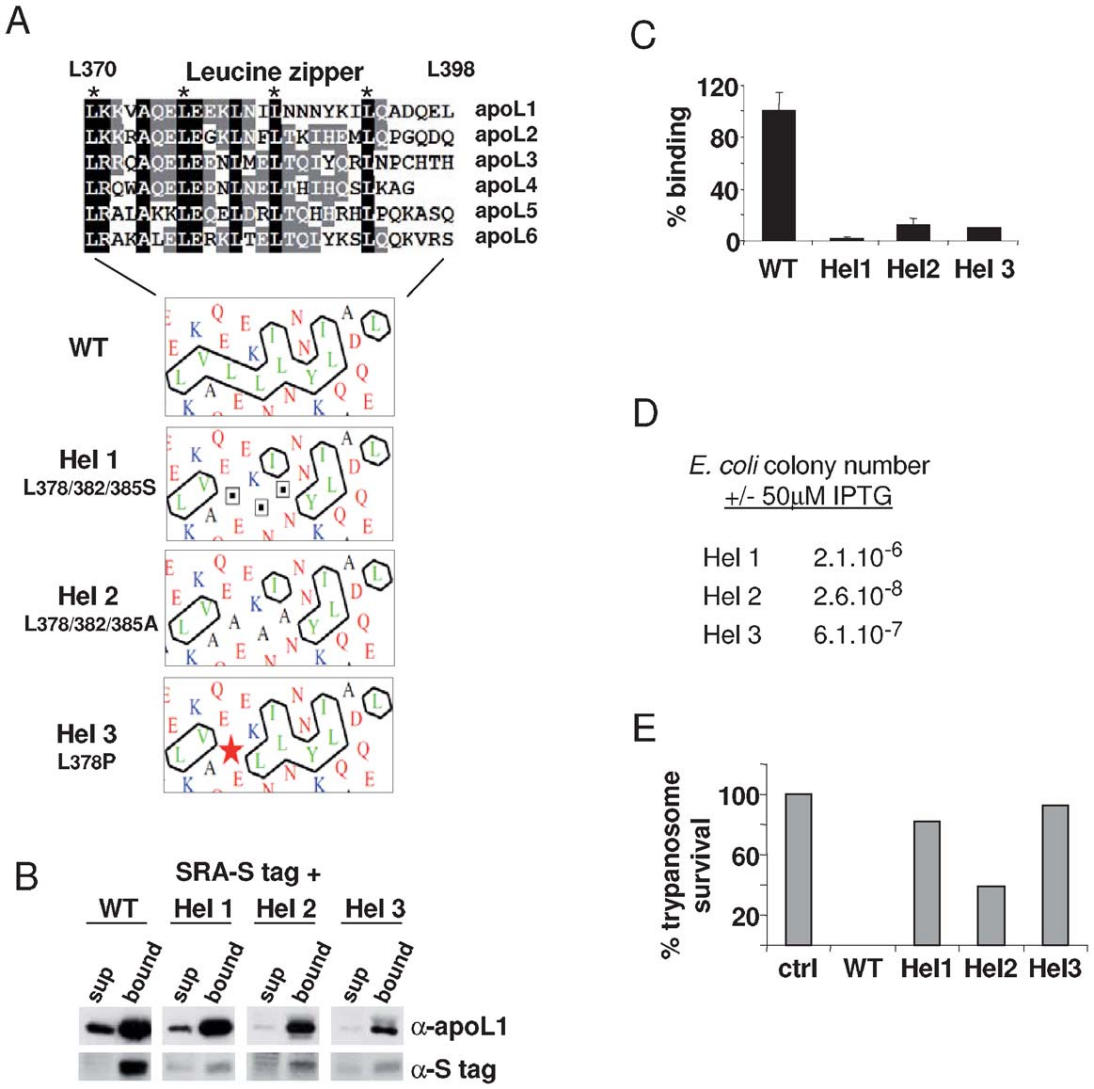
Effects of mutations in the C-terminal leucine zipper of apoL1. **A.** The upper panel shows the sequence alignment of the leucine zipper within the human apoL family. The lower panels show hydrophobic cluster analysis of this region, in WT and various mutant apoL1s. **B.** SRA binding to WT or mutant apoL1s, provided with a C-terminal V5-His6 tag. **C.** Quantification of SRA binding to WT or mutant apoL1s. **D.** Bacterial plating efficiency of various mutant apoL1s. **E.** Trypanolytic potential of various apoL1 variants (ctrl = control).

The C-terminal domain of apoL1, as well as that of the other apoL family members, is characterized by the presence of a leucine zipper (Fig. 3A). Different mutants affecting this zipper were generated (Hel 1: L378/382/385S; Hel 2: L378/382/385A; Hel 3: L378P). As shown in Fig. 3A, in each case hydrophobic cluster analysis predicted a strong reduction of hydrophobic interaction potential [9],[10]. This was confirmed by the prediction of interaction energy using measurement of the mean force potential according to the apoL1-SRA interaction model presented in [1],[11]. This energy was decreased in the mutant sequences (Table 1). As expected from this prediction, SRA interaction was reduced in the three mutants (Fig. 3B,C). However, like in the case of the del 3 variant, these mutants also largely lost their trypanolytic potential although they conserved full pore-forming activity in *E. coli* (Fig. 3D,E). Therefore, conservation of the C-terminal helix appeared to be necessary for the trypanolytic activity of recombinant apoL1.

**Table 1:** Predicted energy of interaction between SRA and the C-terminal domain of various apoL1 mutants.

Interaction energy (kcal/mol)				
WT	Hel1	TKIQ	delTKIQ	delNNNY
−490	−452	−433	−409	−456

The interaction model presented in [1] was used for energy calculation based on the mean force potential [11],[21].

### Identification of Papio apoL1-like proteins possibly involved in trypanolytic activity

**Figure 4:**
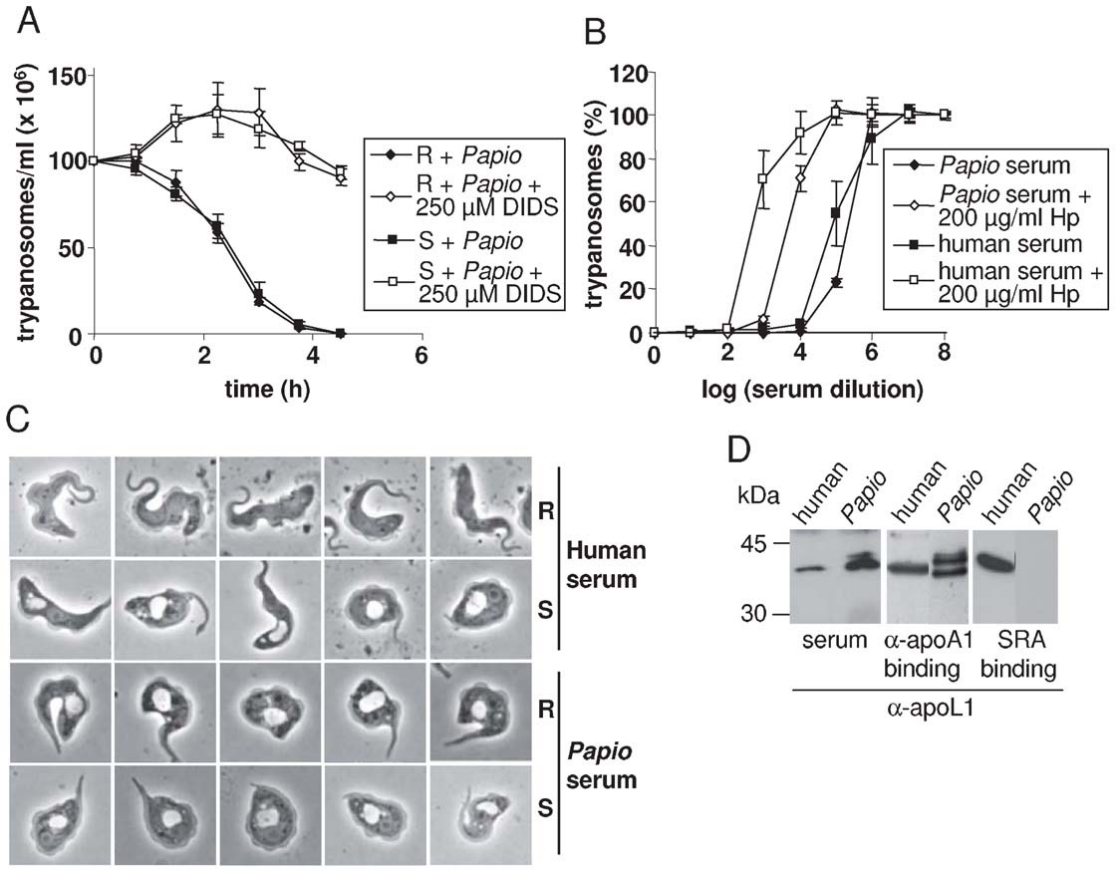
Trypanolytic activity of *Papio cynocephalus*. **A.** Trypanolytic activity of *Papio* serum on NHS-resistant (R) or sensitive (S) clones of *T. b. rhodesiense*, and effect of DIDS on this activity. **B.** Trypanolytic potential of *Papio* and human serum, and effect of haptoglobin (Hp) on this potential. **C.** Phenotype of *T. b. rhodesiense* NHS-resistant (R) or sensitive (S) clones incubated with human or *Papio* serum. **D.** Western blot analysis with anti-apoL1 (goat anti-apoL1 N20 antibody, from Santa Cruz, diluted 1∶100), of human or *Papio* serum and of serum fractions bound to either anti-apoA1 or SRA.

**Figure 5:**
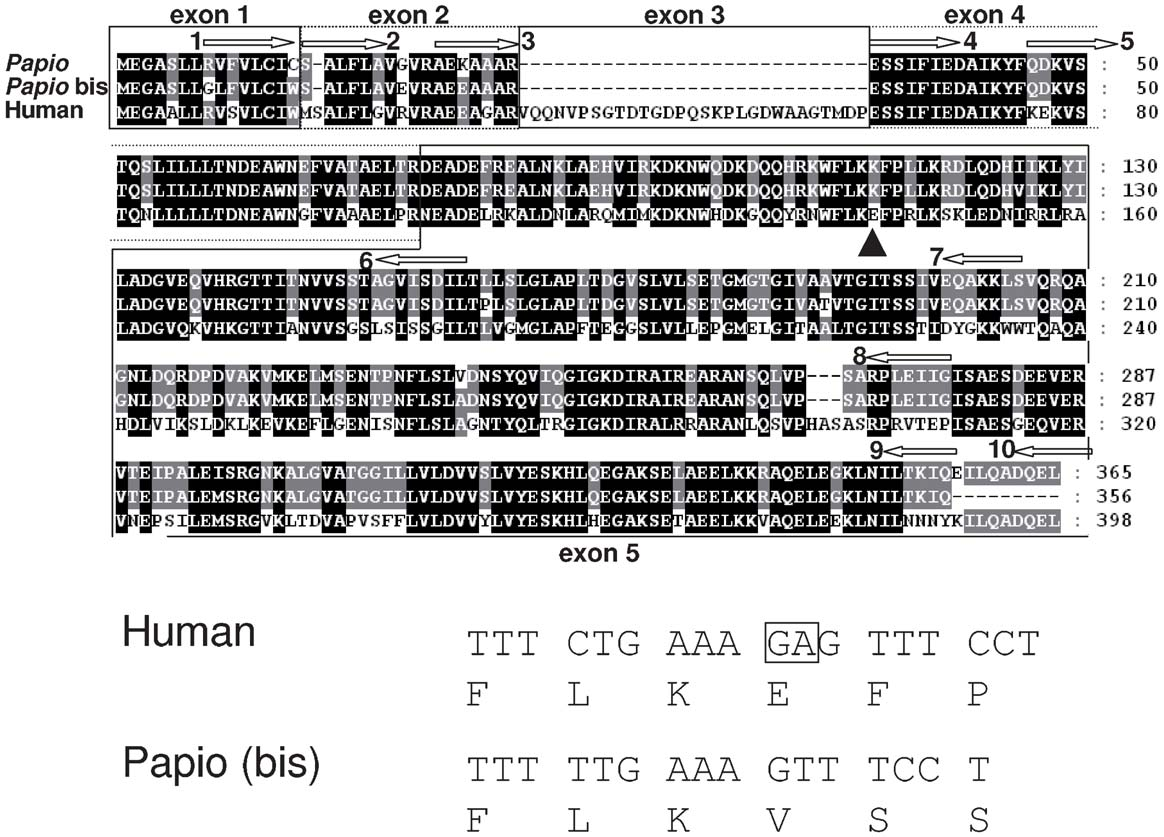
ApoL1-like sequences in *Papio*. Upper panel: Alignment of *Papio* apoL1-like sequences reconstituted from information present in current *Papio* genome databases. The apparent deletion in exon 3 occurs in a region dispensable for the pore-forming activity of human apoL1 [4]. The arrowhead identifies a frameshift predicted in the two apoL1-like genes. Arrows indicate the position and the orientation of the 10 primers used for RT-PCR analysis. Lower panel: Detail of the frameshift: nucleotides of human gene deleted in the *Papio* sequence are boxed.

As shown in Fig. 4A, the serum of *Papio cynocephalus* was equally able to lyze NHS-resistant and -sensitive T. *b. rhodesiense* clones, although it did not affect *T. b. gambiense* (data not shown). As NHS or recombinant apoL1 cannot lyze *T. b. rhodesiense* [1], the *Papio* serum must contain a trypanolytic factor different from apoL1. However, several observations suggest that this factor actually resembles apoL1. First, like apoL1 the *Papio* trypanolytic factor is bound to HDL particles, as *Papio* trypanolytic activity was present in a serum fraction binding to anti-apoA1, the main constituent of HDLs [7]. Second, like apoL1 [4], it was sensitive to inhibition by the anionic channel inhibitor DIDS (Fig. 4A). Third, like apoL1 it was inhibited by competition with an excess of haptoglobin, suggesting its association with Hpr [7] (Fig. 4B). Fourth, the cellular phenotype of trypanosome lysis by *Papio* serum, involving considerable swelling of the lysosome, was indistinguishable from that induced by NHS (Fig. 4C). Finally, proteins isolated from *Papio* serum through binding to anti-apoA1 could be detected by anti-apoL1 antibodies (Fig. 4D). However, as expected considering the lack of resistance of *T. b. rhodesiense* to *Papio* serum, the apoL1-like proteins of *Papio* were unable to bind to SRA (Fig. 4D). In order to evaluate the possible presence of apoL1-like proteins in *Papio* sp., we examined the currently available *Papio anubis* genomic sequence information. As shown in Fig. 5, two apoL1-like sequences were identified, one of which was shorter due to C-terminal truncation. However, from the actual state of information the two genes appeared to be interrupted by frameshift mutations, and we confirmed this frameshift in reverse transcripts of these genes from RNA of either blood cells of *P. cynocephalus* or endometrium of *P. anubis*, using various combinations between 5 forward and 5 reverse primers from different regions (Fig. 5; data not shown). So far we were unable to clone either gene with full coding capacity.

### C-terminal apoL1 mutants inspired by *Papio* sequences kill both *T. b. brucei* and *T. b. rhodesiense*

**Figure 6:**
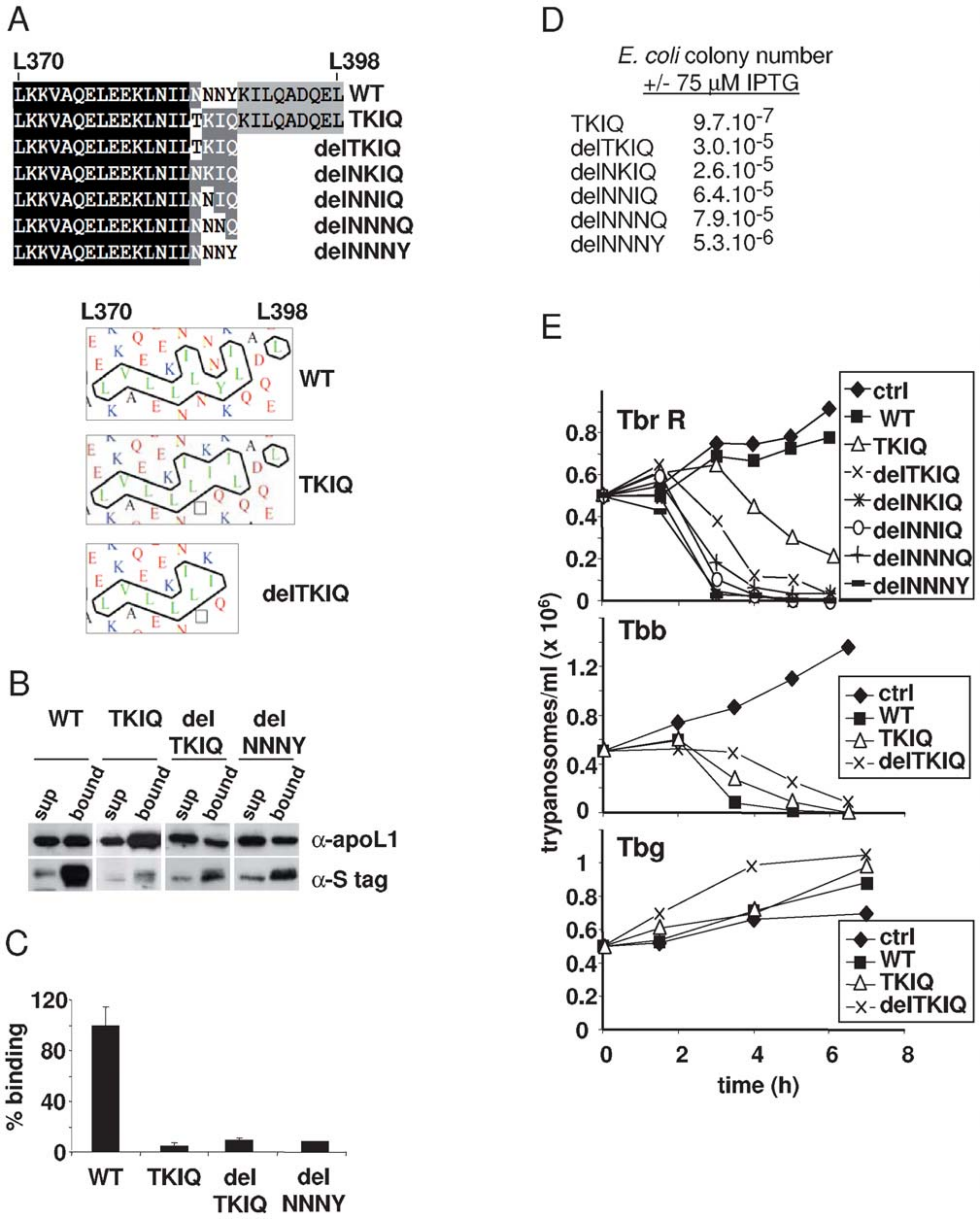
Effects of mutations in the C-terminus of apoL1. **A.** The upper panel shows the sequence of the various mutants. The lower panels show hydrophobic cluster analysis of this region, in WT and two mutants of apoL1. **B.** SRA binding to WT or mutant apoL1s, provided with a C-terminal V5-His6 tag. **C.** Quantification of SRA binding to WT or mutant apoL1s. **D.** Bacterial plating efficiency of various mutant apoL1s. **E.** Trypanolytic activity of various apoL1 variants (30 µg/ml), as determined on NHS-resistant (R) clones of *T. b. rhodesiense*, *T. b. brucei* and *T. b. gambiense* (ctrl = control).

We hypothesized that converting the C-terminal sequence of human apoL1 into those found in the two apoL1-like sequences of *Papio anubis* could impede the interaction of this protein with SRA and confer the capacity to kill *T. b. rhodesiense*. This involved the replacement of the 386–389 sequence NNNY into TKIQ (TKIQ mutant), as well as the same replacement together with the removal of the 9 C-terminal amino acids (delTKIQ mutant) (Fig. 6A). In addition to the latter mutant, we also generated similar mutants where the four C-terminal amino acids of the truncated version were intermediate between *Papio* and human apoL1s (NKIQ, NNIQ, NNNQ, NNNY) (Fig. 6A). These changes did not strongly affect the hydrophobic cluster pattern of the C-terminal helix (Fig. 6A), but they significantly reduced the predicted energy of interaction of apoL1 with SRA according to the model presented in [1] (Table 1). Accordingly, these apoL1 variants lost their capacity to interact with SRA (Fig. 6B,C). All mutants conserved their pore-forming activity in *E. coli* (Fig. 6D). As shown in Fig. 6E, the different *Papio*-like apoL1 variants efficiently killed both *T. b. brucei* and NHS-resistant clones of *T. b. rhodesiense*. However, they were unable to kill *T. b. gambiense*.

**Figure 7:**
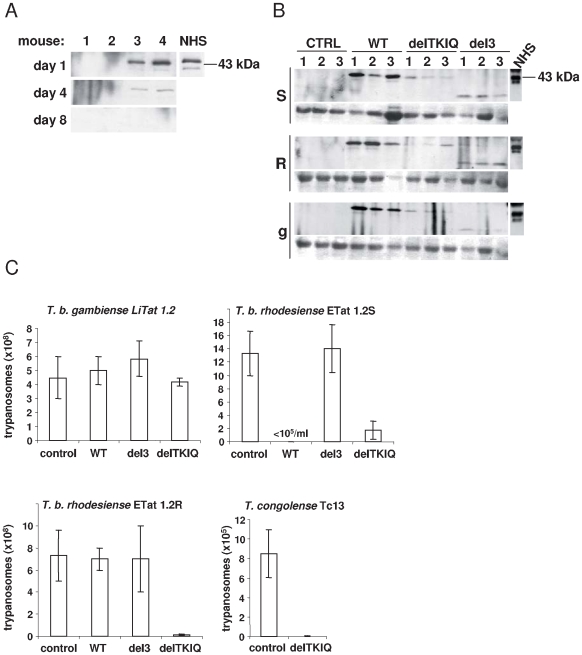
Effect of transient transgenic expression of WT or mutant apoL1 on trypanosome infection in mice. **A.** Detection of WT apoL1 at days 1, 4 and 8 after hydrodynamic injection of the plasmid construct, monitored by incubation of Western blots of mouse serum proteins with rat anti-apoL1 antibodies. Mice 1 and 2 were injected with control (empty) plasmid. The lane labelled NHS shows the result obtained with normal human serum. **B.** Detection of the different apoL1 variants at day 1 post-injection of DNA (CTRL = control empty plamid). The mice whose sera were analyzed here were used for the trypanosome infection experiments reported in panel C (S = *T. b. rhodesiense* ETat 1.2S; R = *T. b. rhodesiense* ETat 1.2R; g = *T. b. gambiense* LiTat 1.2). Normal human serum (NHS) was used for comparison. Loading control by Ponceau red staining of albumin is shown below each panel. **C.** 24 h post-injection of DNA, 10^6^ parasites of the indicated strains were inoculated intraperitoneally into mice. Parasitemia was measured 3 days after parasite inoculation (control: empty plamid).

We evaluated if the *Papio*-like apoL1 variants could exhibit trypanolytic activity in mice as they did *in vitro*. As shown in Fig. 7A, expression of apoL1 can optimally be detected in mice one day after hydrodynamic injection of 10 µg of pCDNA3 plasmid encoding the protein. Similarly, apoL1 variants could be detected one day post-injection of DNA, although the apoL1 mutants appeared to be less expressed than WT apoL1 (Fig. 7B). Intraperitoneal inoculation of 10^6^ trypanosomes from different *T. brucei* subspecies was performed at that day post-DNA injection. Infection by NHS-sensitive *T. b. rhodesiense* ETat 1.2S parasites was inhibited following expression of either WT or delTKIQ apoL1, as determined by the measurement of the parasite number at the peak of parasitaemia (Fig. 7C). As expected, transgenic expression of WT apoL1 did not confer protection against the NHS-resistant *T. b. rhodesiense* clone ETat 1.2R (Fig. 7C). However, mice expressing the delTKIQ apoL1 variant could resist both trypanosome lines (Fig. 7C). Although both WT and delTKIQ apoL1 almost completely erased the first peak of infection by ETat 1.2S parasites, infection reappeared afterwards and in both cases all mice died between days 12 and 15 post-inoculation (data not shown). This infection pattern was also observed for delTKIQ-expressing mice infected with ETat 1.2R. These observations contrast with the complete trypanolysis observed *in vitro* with delTKIQ apoL1, and likely result from the rapid reduction of apoL1 concentration in the transgenic animals (Fig. 7A). The delTKIQ-expressing mice were also able to kill *T. congolense* (Fig. 7C). Therefore, it can be predicted that transgenic cattle constitutively expressing this variant would resist infection by *T. b. brucei*, *T. b. rhodesiense* and *T. congolense*. In contrast, neither WT nor mutant apoL1 conferred protection against *T. b. gambiense* (Fig. 7C).

## Discussion

Our data demonstrate that in *E. coli*, SRA inhibits the pore-forming activity of apoL1 through direct interaction with the C-terminal helix of this protein, and show that apoL1 variants unable to bind SRA through deletion or mutations of this helix could overcome this inhibition. It is particularly interesting to note that the apoL1 variant lacking the original membrane-addressing domain (but containing a bacterial signal peptide to compensate this activity) was still fully inhibited following co-expression of SRA in *E. coli*. This result indicates that the membrane-addressing domain is dispensable for the control of the pore-forming domain by SRA, suggesting that this control does not operate through refolding of the protein. A possible explanation would be that interaction with SRA prevents membrane targeting of apoL1.

Similarly to the situation in *E. coli*, apoL1 variants unable to bind SRA were found to kill NHS-resistant trypanosomes, confirming that the conclusions drawn in *E. coli* regarding the neutralization of the protein by SRA were also valid for trypanosomes. In particular, the fact that in *E. coli* SRA can directly inhibit the activity of apoL1 suggests that in trypanosomes, its effect on apoL1 would not necessarily operate through reorientation of apoL1 trafficking in the cell. However, a clear difference was observed between *E. coli* and trypanosomes. In the latter case, conservation of most of the C-terminal helix was required to keep the lytic potential intact, even for trypanosomes devoid of SRA. Altogether these conclusions essentially confirmed our earlier proposal [1], although our message concerning the truncated apoL1 variant ending at S342 had to be corrected. In [1], we showed data suggesting that this variant, equivalent to the del 3 apoL1 version studied in this work, was able to kill both *T. b. brucei* and *T. b. rhodesiense*. In contrast, in a recent report analyzing the trypanolytic potential of apoL1 expressed transiently in mice following hydrodynamic plasmid transfection, the del 3 variant was found to be no longer trypanolytic [8]. This controversy could be resolved in this study, where we confirmed the last observation. Thus, it would appear that in contrast to what occurs in *E. coli*, in trypanosomes the C-terminal domain of apoL1 is not completely dispensable for the killing activity of the protein. Given the obvious differences between the two biological systems this observation could be explained in many ways. In the *E. coli* system the membrane targeting is direct and entirely intracellular, and like experimentally observed with bacterial colicins the isolated pore-forming domain of apoL1 can exhibit full toxic activity provided a membrane-addressing peptide is present [4]. In trypanosomes the toxin must be taken up through endocytosis, which requires a more complex trafficking pathway, and the target is the lysosomal membrane instead of the plasma membrane. Among other possibilities the C-terminal helix could be required for proper trafficking and/or targeting to the lysosomal membrane, or its absence could result in improper folding of the protein. Regarding the possible influence of the purification procedure on the protein conformation, it should be noted that after reconstitution with lipid particles, recombinant apoL1 was found to kill trypanosomes with efficiency close to that of native apoL1 in normal human serum, suggesting that the full-size recombinant protein is not significantly denatured during purification [4].

Interestingly, it was through inspiration driven by sequence analysis of *Papio* apoL1-like genes that we were able to generate apoL1 variants unable to bind SRA but still able to efficiently kill trypanosomes. As expected, these variants killed NHS-resistant *T. b. rhodesiense* clones as well as NHS-sensitive *T. b. rhodesiense* clones or *T. b. brucei*, like occurs with *Papio* serum. This finding strongly suggests that in *Papio* serum similar apoL1 variants are responsible for the trypanolytic activity. However, we were unable to identify such proteins, as frameshift mutations apparently prevented the possible candidates to be functional. This result was repeatedly observed following RT-PCR amplification of transcripts from either blood cells or endometrium tissue, using various combinations of 5 forward and 5 reverse primers specific to the apoL1-like genes from the *Papio anubis* sequence database. Given our failure to detect any functional *Papio* apoL1-like sequence, we can only speculate about the nature of the trypanolytic factor in these organisms. Among other possibilities, apoL4 could replace apoL1 given the presence of a sequence encoding a putative signal peptide in this gene, as also occurs in humans [12]. However, it should be noted that this apoL4 variant was never detected in human serum so far. Thus, despite the identity between the trypanolytic phenotypes exhibited between *Papio* serum and the apoL1 mutant generated in this work, we cannot exclude that this mutant is without relevance concerning the genuine trypanolytic activity of *Papio sp*. The existence of baboon apoL1 variants able to resist SRA would be consistent with the recent proposal that variations of apoL sequences are frequent at sites of interaction with pathogen proteins [13], although the apoL1 mutations/deletion described here were not described in this report.

As is the case with *Papio* serum [14], the *Papio*-like human apoL1 was unable to kill *T. b. gambiense*. This observation is in keeping with the fact that in this subspecies the mechanism of resistance to apoL1 must be different from that of *T. b. rhodesiense*, as SRA is absent from *T. b. gambiense* [15]. Therefore, it appears that resistance to NHS in *T. b. gambiense* is independent from the C-terminal domain of apoL1.

The results presented in this work are promising in terms of generating transgenic cattle able to resist infection by African trypanosomes in Eastern Africa. Indeed, we show that mice transiently expressing *Papio*-like human apoL1 variants resist not only *T. b. brucei*, but also NHS-resistant clones of *T. b. rhodesiense* and the cattle pathogen *T. congolense*. Moreover, given the sensitivity of *T. evansi* to apoL1 [16] this transgenic cattle should resist *T. evansi* as well, and similar prediction could be proposed for *T. vivax*.

Finally, in view of these results it can be envisaged that understanding the mechanism of resistance of *T. b. gambiense* to NHS would allow us to generate mutant versions of apoL1 also able to kill this parasite.

## Materials and Methods

### Ethics statement

This research was approved by the ethics committee of the Institute for Molecular Biology and Medicine (IBMM). All mice were housed in our pathogen-free facility and the experiments were performed in compliance with the relevant laws and institutional guidelines (license LA2500482).

### Production of recombinant WT and mutant versions of apoL1

The apoL1 coding sequence was amplified by PCR from the pcDNA3.1-apoL1-V5-His_6_ construct [1] with primers creating a 5′ NcoI site and ATG codon upstream from the E28 codon and a 3′ NotI site downstream from the His_6_ tag coding sequence. The PCR fragment was cloned in NcoI and NotI sites of the first polylinker of pCDF-Duet1 expression vector (Novagen). Mutant versions of apoL1 were created by site-directed mutagenesis with Accuprime *Pfx* DNA polymerase (Invitrogen) and verified by sequencing. The complete SRA coding sequence was obtained by PCR from pTSA-Rib-SRA [5] with primers creating a 5′ Ecl136II and 3′ SalI sites. The DNA fragment was cloned in frame with the S-tag coding sequence in EcoRV and XhoI sites of the second polylinker of pCDF-Duet1 vector and pCDF-Duet apoL1-V5-His_6_ construct.

The plasmid constructs were transfected into *E. coli* BL21(DE3) and expression of recombinant proteins was induced from an exponentially growing culture by 1 mM isopropyl β-D-1-thiogalactopyranoside (IPTG) overnight at 37°C. Bacteria were resuspended in 50mM Tris-HCl (pH 9.2) and lysed by two passages in French Press. Cell debris were removed by centrifugation at 5000g for 10 min, and inclusion bodies were recovered from supernatant by centrifugation at 16,000g for 15 min. Purification was performed from inclusion bodies dissolved in 6 M guanidium chloride, 150 mM NaCl, 50 mM Tris-HCl (pH 8.0). Solubilized proteins were incubated for 1 h with Ni-NTA beads (Qiagen), and the bound proteins were extensively washed with 4 M guanidium chloride, 50 mM Tris-HCl (pH 7.5), 150 mM NaCl, 20 mM imidazole, before elution with 4 M guanidium chloride in 0.2 M acetic acid. After dialysis against 20 mM acetic acid, the proteins were concentrated (Vivaspin, Sartorius). Purity and concentration were estimated by SDS-PAGE and Coomassie Blue staining.

### Pore-forming activity in bacteria

Cultures of BL21(DE3) strains transfected with pCDF-Duet1 constructs were grown at 37°C in LB containing 1% glucose and 50 µg/ml streptomycin from freshly plated colonies until optical density at 600nm (OD600) reached 0.7 to 0.8. Serial dilutions were plated onto LB containing 50 µg/ml streptomycin and either 1% glucose (non-induced control) or 50 to 75 µM IPTG (induction of recombinant proteins). Usually the pH of LB was 6.0, but the effect of raising the pH to 7.5 was evaluated. Colonies were counted after overnight incubation at 37°C and results were expressed as the *ratio* of colonies with and without IPTG induction. These results reflect the pore-forming activity of apoL1 [4].

### Copurification of apoL1 and SRA from bacteria

Cultures of BL21(DE3) strains transfected with pCDF-Duet1 constructs were grown at 37°C in LB containing 50 µg/ml streptomycin and 1% glucose from freshly plated colonies untill OD600 reached 0.7 to 0.8. Cultures were centrifuged and bacteria pellet was resuspended in fresh medium without glucose and distributed in 3 flasks to perform induction and copurification in triplicate. Expression of recombinant proteins was induced by addition of 1mM IPTG overnight at 20°C and 80 rpm. Cell density was measured by OD600 and bacteria were centrifuged and resuspended in (10% of culture volume×OD600) in cold hypotonic buffer (50 mM MES pH 6.0 or pH 7.0) containing EDTA-free protease inhibitors (Roche). Bacteria were lysed by Fast Prep 24 (MP Biomedicals) with 1/10 volume of glass beads (Lysing Matrix B, MP Biomedicals), for 3×30 s at 6 m.s^−1^. Cell lysate was complemented by 0.6M NaCl, 1% Triton X100, 20 mM imidazole, and vortexed vigorously. Cell debris were pelleted by centrifugation for 15 min at 16,000 g, and the supernatant was applied onto Ni-NTA beads (Qiagen) equilibrated in the same buffer, for 2 h at 4°C. Fig. S1 shows a comparative evaluation of the yield of the different apoL1 variants and SRA in the supernatants. After binding the beads were washed with 20 volumes of beads with cold binding buffer and the bound proteins were eluted with 2 volumes of SDS-PAGE sample buffer. Supernatant and bound fractions were analyzed by western blotting. ApoL1 was revealed with anti-recombinant apoL1 rat serum and peroxydase conjugated anti-rat antibody. SRA-Stag was detected by anti-Stag monoclonal antibody (Novagen) and peroxydase conjugated anti-mouse antibody. Peroxydase activity was revealed by ECL (Western Lighting Chemiluminescence Reagent PLUS, Perkin Elmer). Signals were quantified in supernatant and bound fractions using ImageQuant TL Software (GE Healthcare). Since only a fraction of each supernatant and elution was loaded onto the gel, the values obtained were reported to the total volume of lysis or elution (“supernatant” and “bound” lanes respectively). ApoL1 and SRA binding values to nickel were calculated separately and the binding of SRA to apoL1 was expressed as “SRA binding *versus* apoL1 binding” ratio. The ratio obtained for SRA binding to WT apoL1 was considered as 100%.

### Parasites

*Trypanosoma brucei brucei* 328-114 [17] were grown in HMI9 supplemented with 10% foetal bovine serum, 10% Serum Plus [18] at 37°C in 5% CO_2_. *T. b. rhodesiense* ETat 1.2 NHS-resistant (R) and NHS-sensitive (S) clones and *T. b. gambiense* LiTat 1.2 were grown in IMDM medium supplemented with 20% foetal bovine serum [19].

### *In vitro* trypanolysis assays

The parasites were diluted at a final concentration of 5.10^5^ cells/ml, and recombinant WT or mutant apoL1, in 20 mM acetic acid, was added to reach 10 to 30 µg/ml. Dilution of the parasite culture medium never exceeded 15%, and in control samples without apoL1 the medium was similarly diluted with 20 mM acetic acid. As no attempt was made to reconstitute these proteins into HDL particles, the formation of aggregates is likely. Living parasites were counted in duplicate under the microscope. When lysis was not complete after 8 h, parasites were counted in duplicate after 24 h incubation and cell survival was expressed as % of control. Lysis experiments with human and *Papio* serum, as well as imaging of living immobilised trypanosomes, were performed as described in [20].

### Sequence analysis

Alignments were performed by CLUSTALW software and edited with GeneDoc (http://www.psc.edu/biomed/genedoc). Hydrophobic Cluster Analysis (HCA) was used to compare the proteins at 2D level [9],[10] (http://mobyle.rpbs.univ-paris-diderot.fr/cgi-bin/portal.py?form=HCA).

### Energy calculation based on mean force potential

To calculate the energy of interaction, we used an approach based on a non-local energy between peptides [21]. The mean force potentials used here are based on 500 proteins, using 7 different atomic types, which are polar and nonpolar hydrogens (H), Csp2 and Csp3, O, S and N. Different functions have been evaluated for all the possible atomic pairs. These energy functions have been estimated for distances going from 0.0 to 10.0 Å with a 0.1 Å precision.

### Transient expression in mice

The hydrodynamic transfection of mice was performed according to the method described in [22]. Briefly, 2 ml of saline solution containing 10 µg of pcDNA3-1 or pcDNA3-1 WT apoLI-V5His/del3-V5His/delTKIQ-V5His were injected in less than 8 seconds in the vein of the tail of 8 weeks-old BALB/c female mice. At day 1, an aliquot of blood was analyzed by Western blot for expression of apoL1 and mice were injected intraperitoneally with 10^6^ trypanosomes.

### Accession number

Apolipoprotein L-I, NM_003661.

## Supporting Information

Figure S1 Expression of apoL1 variants and SRA in *E. coli*. Western blots of serial dilutions of *E. coli* soluble extracts were incubated with rat anti-apoL1 or rabbit anti-S tag antibodies. As shown on the right, the relative efficiency of apoL1-SRA binding is unrelated to either protein yield.
(0.99 MB EPS)
